# A pyrenoid-based CO_2_-concentrating mechanism can be effective and efficient even under scenarios of high membrane CO_2_ permeability

**DOI:** 10.1093/plphys/kiaf316

**Published:** 2025-08-12

**Authors:** Chenyi Fei, Ned S Wingreen, Martin C Jonikas

**Affiliations:** Lewis-Sigler Institute for Integrative Genomics, Princeton University, Princeton, NJ 08544, USA; Department of Molecular Biology, Princeton University, Princeton, NJ 08544, USA; Lewis-Sigler Institute for Integrative Genomics, Princeton University, Princeton, NJ 08544, USA; Department of Molecular Biology, Princeton University, Princeton, NJ 08544, USA; Department of Molecular Biology, Princeton University, Princeton, NJ 08544, USA; Howard Hughes Medical Institute, Princeton University, Princeton, NJ 08544, USA

## Abstract

Modeling demonstrates that pyrenoid based CO₂-concentrating mechanisms can be effective and efficient even when membrane permeability to CO₂ is high.

Dear Editor,

There is currently substantial interest in engineering CO_2_-concentrating mechanisms (CCMs) into C3 crops to increase yields in the context of climate change. Kaste et al. (2024) suggest that under scenarios of high membrane permeability to CO_2_, pyrenoid-based CCMs (pCCMs) would be too energetically inefficient to enhance the growth of vascular plants. We believe this conclusion is incorrect. First, we note that energetic efficiency is likely not the primary value of most CCMs currently found in plants: the C4 CCM enables net carbon assimilation under conditions where daily photorespiration plus respiration would otherwise exceed carboxylation, such as high temperature or submergence (Sage 2004), and enhances water-use and nitrogen-use efficiencies (Vogan and Sage 2011). Second, Kaste et al. argue that previous modeling efforts, including ours (Fei et al. 2022), overestimated the energetic efficiency of pCCMs by assuming a CO_2_ membrane permeability on the low end of the experimentally measured range. However, here we show that our pCCM model can exhibit energetically efficient operation even in scenarios of high membrane permeability. Our results reaffirm the theoretical feasibility of enhancing C3 crop yields and climate resilience by endowing them with a pCCM.

The claim by Kaste et al. that pCCM performance suffers under high membrane permeability led us to revisit our model with specific attention to the impact of barriers that limit CO_2_ escape. Diffusion barriers are known to be essential for the optimal function of a pCCM (Fridlyand 1997; Toyokawa et al. 2020) as they help retain CO_2_ within the pyrenoid, thereby enhancing the efficacy and efficiency of carbon fixation.

Pyrenoids are surrounded by CO_2_ diffusion barriers made of starch (Toyokawa et al. 2020), multiple stacked membranes (Sager and Palade 1957), or protein shells (Nam et al. 2024; Shimakawa et al. 2024), which are thought to slow the escape of CO_2_ (Fridlyand 1997), resulting in a high concentration of CO_2_ in the pyrenoid and reducing futile cycling. In the leading model alga *Chlamydomonas reinhardtii*, both the thylakoid membrane sheets and the pyrenoid starch sheath could serve as CO_2_ diffusion barriers. While an earlier study suggested that a starchless mutant had normal pCCM performance in air, the phenotype was not compared to the appropriate parental strain (Villarejo et al. 1996). A more recent study by Toyokawa et al. (2020) found that mutants with a thinner starch sheath than wild-type strains (*sta2-1*) or lacking a starch sheath (*sta11-1*) display decreased pCCM efficacy at very low CO_2_, which supports the role of the starch sheath as a CO_2_ leakage barrier.

Here, we systematically explored the impact of membrane permeability and CO_2_ leakage barriers on the energetic efficiency of the pCCM by using our well-characterized model of the *Chlamydomonas* pCCM (Fei et al. 2022). While our model is at the chloroplast level and does not fully account for the processes outside the organelle, it successfully recapitulates all known *Chlamydomonas* pCCM-deficient phenotypes (Fei et al. 2022). Like the Kaste model, our model seeks to capture the key CO_2_ and HCO_3_^−^ reaction and transport processes in a chloroplast. However, our model differs from the Kaste model in several ways; most notably, our model explicitly depicts thylakoid tubules that allow transport of CO_2_ and HCO_3_^−^ radially into the pyrenoid and separately represents a CO_2_ leakage barrier. We think that this geometry allows our model to capture HCO_3_^−^ transport and CO_2_ leakage more faithfully than the Kaste model, and, together with specific parameter choices, these differences may explain why our model is able to achieve better performance.

Our previous modeling results demonstrated that 2 distinct carbon uptake modes are feasible for a pCCM under air-level CO_2_: an active mode and a passive mode (Fei et al. 2022). The active mode relies on an HCO_3_^−^ pump across the chloroplast membrane to accumulate HCO_3_^−^ in the chloroplast stroma (Fig. 1). By contrast, the passive mode lacks an HCO_3_^−^ pump and instead employs a stromal carbonic anhydrase to capture CO_2_ that diffuses in passively across the chloroplast envelope, thereby creating a high concentration of HCO_3_^−^ in the high-pH stroma (Fig. 2). In both the active and passive uptake modes, stromal HCO_3_^−^ subsequently diffuses across the thylakoid membrane into the thylakoid lumen. Within the portion of the thylakoid lumen that traverses the pyrenoid matrix, a carbonic anhydrase converts HCO_3_^−^ to CO_2_, which diffuses out of the lumen, increasing the concentration of CO_2_ in the matrix.

**Figure 1.**
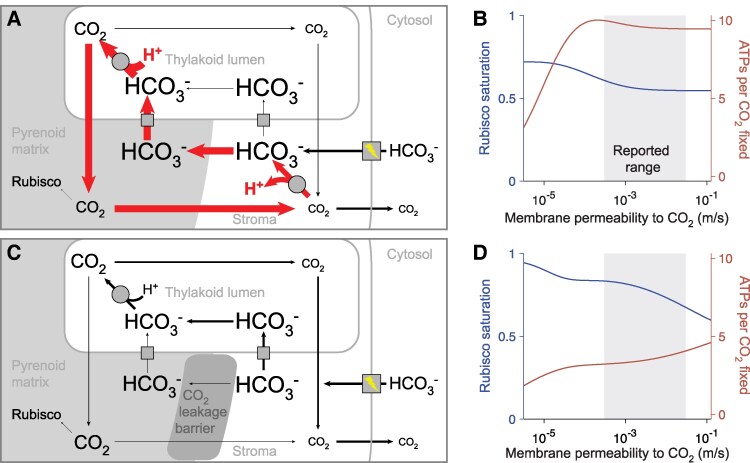
A pCCM employing active carbon uptake can be effective and efficient even with high membrane permeability. We used a spherically symmetrical reaction-diffusion model (Fei *et al*. 2022) to simulate the pCCM. The chloroplast consists of 3 compartments with different pH values: a pyrenoid matrix in the center (pH = 8), a surrounding stroma (pH = 8), and thylakoids traversing the matrix and the stroma (pH = 6), and is surrounded by a well-mixed cytosol (pH = 7). We consider 2 inorganic carbon (Ci) species, CO_2_ and HCO_3_^−^, with font size proportional to their average molecular concentrations in each compartment. CO_2_ diffuses across all membranes and HCO_3_^−^ diffuses across the thylakoid membranes via passive channels (gray squares). In the active carbon uptake mode, HCO_3_^−^ is transported across the chloroplast membrane by active pumps (gray square with lightning symbol). The interconversion between CO_2_ and HCO_3_^−^ is catalyzed by 2 carbonic anhydrases (gray circles), one uniformly distributed in the stroma and another localized to the pyrenoid portion of the thylakoid lumen. CO_2_ is fixed by Rubisco in the pyrenoid matrix. Arrows indicate the net Ci fluxes in the processes described above, with arrow width proportional to flux. **A)** We used our model to simulate a configuration lacking a CO_2_ leakage barrier around the pyrenoid matrix and including a stromal carbonic anhydrase. This configuration leads to a substantial flux through a futile cycle (indicated by thick red arrows), contributing to poor energy efficiency of the pCCM. **B)** Graph indicating Rubisco saturation, defined as the ratio of Rubisco's CO_2_ fixation flux to its saturated maximum, and ATPs per CO_2_ fixed, shown for varying membrane permeabilities *k*_c_ to CO_2_, for the model shown in **A)**. **C)** Removing the stromal carbonic anhydrase and adding an effective barrier to pyrenoidal CO_2_ leakage, such as a starch sheath and/or thylakoid membrane sheets, decrease futile cycling, increase the concentration of CO_2_ in the matrix, and enhance CO_2_ fixation. **D)** Rubisco saturation and ATPs per CO_2_ fixed for varying membrane permeabilities *k*_c_ to CO_2_ for the model shown in **C)**. Simulation parameters are listed in Supplementary Table S1.

**Figure 2.**
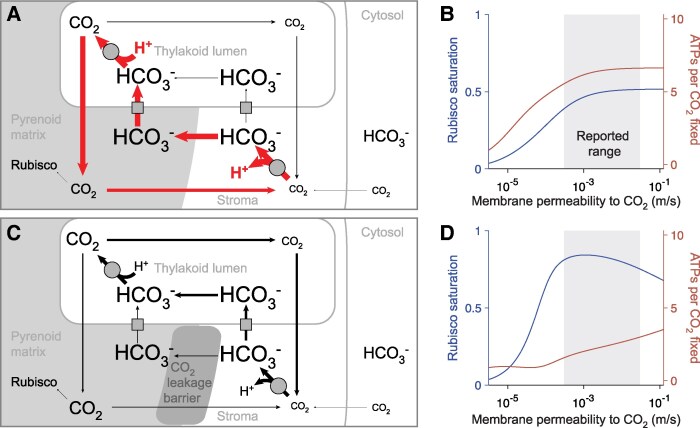
A pCCM employing passive carbon uptake can be effective and efficient even with high membrane permeability. **A)** We simulate passive uptake in the absence of a CO_2_ leakage barrier by using the configuration shown in Fig. 1A without the active HCO_3_^−^ pump on the chloroplast envelope. Again, the absence of a CO_2_ leakage barrier around the pyrenoid matrix leads to a substantial flux through a futile cycle (indicated by thick red arrows), contributing to poor energy efficiency of the pCCM. **B)** Rubisco saturation and ATPs per CO_2_ fixed for varying membrane permeabilities *k*_c_ to CO_2_ for the model shown in **A)**. **C)** Adding an effective barrier to pyrenoidal CO_2_ leakage, such as a starch sheath and/or thylakoid membrane sheets, decreases futile cycling, increases the concentration of CO_2_ in the matrix, and enhances CO_2_ fixation. **D)** Rubisco saturation and ATPs per CO_2_ fixed for varying membrane permeabilities *k*_c_ to CO_2_ for the model shown in **C)**. Simulation parameters are listed in Supplementary Table S1.

For each of these 2 operational modes of a pCCM, we conducted a series of simulations of our model, varying the CO_2_ permeability *k*_c_ of the thylakoid and chloroplast membranes (Figs. 1, B and D, and 2, B and D). Following our previous work, we use 2 metrics, Rubisco saturation by CO_2_ and ATPs per CO_2_ fixed, to characterize, respectively, the efficacy and energetic efficiency of the modeled pCCM (Fei et al. 2022).

We start by considering the active HCO_3_^−^ uptake mode (Fig. 1). While it is possible in principle to increase the rate of HCO_3_^−^ pumping indefinitely to fully saturate Rubisco, this would incur an excessively high energy cost. Indeed, our simulation results at varying HCO_3_^−^ pumping rates show that a pumping rate larger than 10^−4^ m/s consistently gives rise to poor energy efficiency across a wide range of conditions (Supplementary Fig. S1). Notably, Kaste et al. used a baseline first-order pumping rate of 8.5 × 10^−4^ m/s, which could contribute to the low efficiency observed in their model. Here, we choose a pumping rate of 5 × 10^−5^ m/s to achieve a balanced performance of both efficacy and energy efficiency.

To underscore the importance of accurate modeling of a barrier to CO_2_ diffusion out of the pyrenoid matrix, we first characterized our model with this barrier omitted (Fig. 1, A and B). In this configuration, Rubisco saturation is only slightly above 50% and decreases with increasing membrane CO_2_ permeability *k*_c_ as more CO_2_ diffuses out of the chloroplast (Fig. 1B). The energy cost is high (9 to 10 ATPs per CO_2_ fixed) throughout the range of experimentally reported *k*_c_ values (10^−5^ m/s to 3 × 10^−2^ m/s; Fig. 1B, gray region) (Gutknecht et al. 1977; Sültemeyer and Rinast 1996; Missner et al. 2008; Hopkinson et al. 2011), due in large part to a futile cycle where more than 90% of concentrated CO_2_ leaks out from the pyrenoid (Fig. 1A, thick red arrows). Specifically, stromal HCO_3_^−^ diffuses into the low-pH thylakoid lumen and consumes 1 proton during its conversion to CO_2_ by the thylakoid CA. This CO_2_ can readily diffuse from the thylakoids into the pyrenoid matrix, but without a barrier, most of the CO_2_ molecules escape the matrix without being fixed by Rubisco. The escaping CO_2_ is then captured by the stromal CA and converted back to HCO_3_^−^, releasing 1 proton into the high-pH stroma. Consequently, the net effect of this futile cycling is the transfer of 1 proton from the low-pH lumen to the high-pH stroma for each leaked CO_2_, which costs the amount of energy necessary to pump the protons back to maintain the intercompartmental pH difference.

Disrupting this futile cycle by removing the stromal carbonic anhydrase and adding a diffusion barrier with low permeability drastically reduces CO_2_ leakage from the matrix to the stroma (Fig. 1, C and D). We evaluated the performance of the modeled CCM across a range of diffusion barrier permeabilities and found that values below 10^−4^ m/s essentially render the barrier impermeable (Supplementary Fig. S1). Although the diffusion barrier permeability has not been directly measured in *Chlamydomonas*, previous studies show that the pyrenoid is surrounded by stacks of 10 or more thylakoid membranes (Engel et al. 2015; Hennacy et al. 2024), which together can reduce the effective permeability to 1/10 that of a single membrane. Given that the reported CO_2_ permeability of a single membrane ranges from 10^−5^ m/s to 3 × 10^−2^ m/s, an overall barrier permeability of 10^−4^ m/s is biologically plausible. Moreover, a diffusion barrier permeability on the order of 10^−4^ m/s allows us to fit the model to previous Ci affinity measurements (Supplementary Fig. S2) (Toyokawa et al. 2020). Thus, we use a diffusion barrier permeability of 10^−4^ m/s, which could correspond to several membranes stacked together or a starch sheath barrier. With the diffusion barrier in place, the active mode of the pCCM becomes both effective and efficient, achieving a Rubisco saturation of ∼80% with an energy cost of 3 to 4 ATPs per CO_2_ fixed over the range of measured membrane permeability conditions (Fig. 1D, gray region).

We obtained similar results for the passive CO_2_ uptake mode (Fig. 2). Rubisco saturation increased with *k*_c_ as this mode relies on the diffusive influx of CO_2_ across the chloroplast membrane (Fig. 2, B and D). When we omitted a diffusion barrier (Fig. 2A), Rubisco saturation remained below roughly 50% across the range of experimentally reported values (Fig. 2B), and the energy cost was high (6 to 7 ATPs per CO_2_ fixed; Fig. 2B) due to futile cycling caused by CO_2_ leakage from the pyrenoid (Fig. 2A, thick red arrows). The performance of the passive mode was greatly enhanced with the addition of a diffusion barrier and became largely independent of *k*_c_ within the range of experimentally reported values. In this case, a pCCM with passive CO_2_ uptake achieved ∼80% Rubisco saturation with an energy cost of 2 to 3 ATPs per CO_2_ fixed.

The passive-uptake pCCM's energetic cost of 2 to 3 ATPs per CO_2_ fixed is comparable to the expected energetic cost of photorespiration in the absence of a CCM. The energetic cost of photorespiration per oxygenation reaction is estimated to be 3.5 ATP and 2 NADH equivalents (Walker et al. 2016), equivalent to ∼8.1 ATPs if we assume 1 NADH is approximately worth 2.3 ATPs (Rich 2003). The ratio of oxygenation to carboxylation reactions varies depending on the organism and environment; if one assumes a ratio of 2:5 (Sharkey 1988), corresponding to 1 oxygenation reaction per 2 net CO_2_ fixed, this translates to an approximate photorespiratory cost of 4 ATPs per net CO_2_ fixed. Thus, the energetic costs of either the active or passive CO_2_ uptake in pCCMs can theoretically be comparable to the energetic costs of photorespiration, which the pCCMs largely obviate while enhancing CO_2_ fixation.

In summary, our results indicate that an effective and energy-efficient pCCM can operate in air with active or passive CO_2_ uptake, even in scenarios of high membrane CO_2_ permeability. Energy-efficient operation requires a sufficient barrier to CO_2_ diffusion out of the pyrenoid matrix such as thylakoid membranes, a starch sheath, or a protein barrier. We suspect that the observations of low energy efficiency in Kaste et al. (2024) could be due to the limitations of how that model represents inorganic carbon diffusion into and out of the pyrenoid. Our results underscore the importance of ongoing efforts to engineer CO_2_ diffusion barriers (Atkinson et al. 2024) as part of the broader effort to engineer pyrenoids into vascular plants.

## Supplementary Material

kiaf316_Supplementary_Data

## Data Availability

All data generated in this study are deposited in the Zenodo repository at https://doi.org/10.5281/zenodo.14617701 Simulation codes used to generate the data are available on GitHub at https://github.com/f-chenyi/Chlamydomonas-CCM.

## Supplementary data

The following materials are available in the online version of this article.

**Supplementary Figure S1.** The performance of a pCCM depends on the permeability of the CO_2_ leakage barrier around the pyrenoid.

**Supplementary Figure S2.** Our model of the pCCM is consistent with the inorganic carbon (Ci) affinity of wild-type *Chlamydomonas* cells and starch-deficient mutants measured in previous experiments.

**Supplementary Table S1.** Simulation parameters for the model of the pCCM.

## Dive Curated Terms

The following phenotypic, genotypic, and functional terms are of significance to the work described in this paper:

NADH CHEBI: CHEBI:16908

ATP CHEBI: CHEBI:15422

## References

[kiaf316-B1] Atkinson N, Stringer R, Mitchell SR, Seung D, McCormick AJ. SAGA1 and SAGA2 promote starch formation around proto-pyrenoids in *Arabidopsis* chloroplasts. Proc Natl Acad Sci U S A. 2024:121(4):e2311013121. 10.1073/pnas.2311013121 38241434 PMC10823261

[kiaf316-B2] Engel BD, Schaffer M, Cuellar LK, Villa E, Plitzko JM, Baumeister W. Native architecture of the *Chlamydomonas* chloroplast revealed by in situ cryo-electron tomography. eLife. 2015:4:e04889. 10.7554/eLife.04889 25584625 PMC4292175

[kiaf316-B3] Fei C, Wilson AT, Mangan NM, Wingreen NS, Jonikas MC. Modelling the pyrenoid-based CO_2_-concentrating mechanism provides insights into its operating principles and a roadmap for its engineering into crops. Nat Plants. 2022:8(5):583–595. 10.1038/s41477-022-01153-7 35596080 PMC9122830

[kiaf316-B4] Fridlyand LE . Models of CO_2_ concentrating mechanisms in microalgae taking into account cell and chloroplast structure. Biosystems. 1997:44(1):41–57. 10.1016/S0303-2647(97)00042-7 9350356

[kiaf316-B5] Gutknecht J, Bisson MA, Tosteson FC. Diffusion of carbon dioxide through lipid bilayer membranes: effects of carbonic anhydrase, bicarbonate, and unstirred layers. J Gen Physiol. 1977:69(6):779–794. 10.1085/jgp.69.6.779 408462 PMC2215341

[kiaf316-B6] Hennacy JH, Atkinson N, Kayser-Browne A, Ergun SL, Franklin E, Wang L, Eicke S, Kazachkova Y, Kafri M, Fauser F, et al SAGA1 and MITH1 produce matrix-traversing membranes in the CO_2_-fixing pyrenoid. Nat Plants. 2024:10(12):2038–2051. 10.1038/s41477-024-01847-0 39548241 PMC11649565

[kiaf316-B7] Hopkinson BM, Dupont CL, Allen AE, Morel FMM. Efficiency of the CO_2_-concentrating mechanism of diatoms. Proc Natl Acad Sci U S A. 2011:108(10):3830–3837. 10.1073/pnas.1018062108 21321195 PMC3054024

[kiaf316-B8] Kaste JAM, Walker BJ, Shachar-Hill Y. Reaction-diffusion modeling provides insights into biophysical carbon-concentrating mechanisms in land plants. Plant Physiol. 2024:196(2):1374–1390. 10.1093/plphys/kiae324 38857179 PMC11444298

[kiaf316-B9] Missner A, Kügler P, Saparov SM, Sommer K, Mathai JC, Zeidel ML, Pohl P. Carbon dioxide transport through membranes. J Biol Chem. 2008:283(37):25340–25347. 10.1074/jbc.M800096200 18617525 PMC2533081

[kiaf316-B10] Nam O, Musiał S, Demulder M, McKenzie C, Dowle A, Dowson M, Barrett J, Blaza JN, Engel BD, Mackinder LCM. A protein blueprint of the diatom CO_2_-fixing organelle. Cell. 2024:187(21):5935–5950.e18. 10.1016/j.cell.2024.09.025 39368476

[kiaf316-B11] Rich PR . The molecular machinery of Keilin's respiratory chain. Biochem Soc Trans. 2003:31(6):1095–1105. 10.1042/bst0311095 14641005

[kiaf316-B12] Sage RF . The evolution of C_4_ photosynthesis. New Phytol. 2004:161(2):341–370. 10.1111/j.1469-8137.2004.00974.x 33873498

[kiaf316-B13] Sager R, Palade GE. Structure and development of the chloroplast in *Chlamydomonas*: I. The normal green cell. J Biophys Biochem Cytol. 1957:3(3):463–488. 10.1083/jcb.3.3.463 13438931 PMC2224040

[kiaf316-B14] Sharkey TD . Estimating the rate of photorespiration in leaves. Physiol Plant. 1988:73(1):147–152. 10.1111/j.1399-3054.1988.tb09205.x

[kiaf316-B15] Shimakawa G, Demulder M, Flori S, Kawamoto A, Tsuji Y, Nawaly H, Tanaka A, Tohda R, Ota T, Matsui H, et al Diatom pyrenoids are encased in a protein shell that enables efficient CO_2_ fixation. Cell. 2024:187(21):5919–5934.e19. 10.1016/j.cell.2024.09.013 39357521

[kiaf316-B16] Sültemeyer D, Rinast K-A. The CO_2_ permeability of the plasma membrane of *Chlamydomonas reinhardtii*: mass-spectrometric ^18^O-exchange measurements from ^13^C^18^O_2_ in suspensions of carbonic anhydrase-loaded plasma-membrane vesicles. Planta. 1996:200(3):358–368. 10.1007/BF00200304

[kiaf316-B17] Toyokawa C, Yamano T, Fukuzawa H. Pyrenoid starch sheath is required for LCIB localization and the CO_2_-concentrating mechanism in green algae. Plant Physiol. 2020:182(4):1883–1893. 10.1104/pp.19.01587 32041908 PMC7140920

[kiaf316-B18] Villarejo A, Martinez F, del Pino Plumed M, Ramazanov Z. The induction of the CO_2_ concentrating mechanism in a starch-less mutant of *Chlamydomonas reinhardtii*. Physiol Plant. 1996:98(4):798–802. 10.1111/j.1399-3054.1996.tb06687.x

[kiaf316-B19] Vogan PJ, Sage RF. Water-use efficiency and nitrogen-use efficiency of C_3_-C_4_ intermediate species of *Flaveria* Juss. (Asteraceae). Plant Cell Environ. 2011:34(9):1415–1430. 10.1111/j.1365-3040.2011.02340.x 21486309

[kiaf316-B20] Walker BJ, VanLoocke A, Bernacchi CJ, Ort DR. The costs of photorespiration to food production now and in the future. Annu Rev Plant Biol. 2016:67(1):107–129. 10.1146/annurev-arplant-043015-111709 26865340

