# Supplementary material for: A pyrenoid-based CO2-concentrating mechanism can be effective and efficient even under scenarios of high membrane CO2 permeability
Source: Plant Physiol. 2025 Aug 12;198(4):kiaf316. doi: 10.1093/plphys/kiaf316 (PMC12343021; doi:10.1093/plphys/kiaf316)
Supplement: kiaf316_Supplementary_Data [file kiaf316_supplementary_data.pdf]

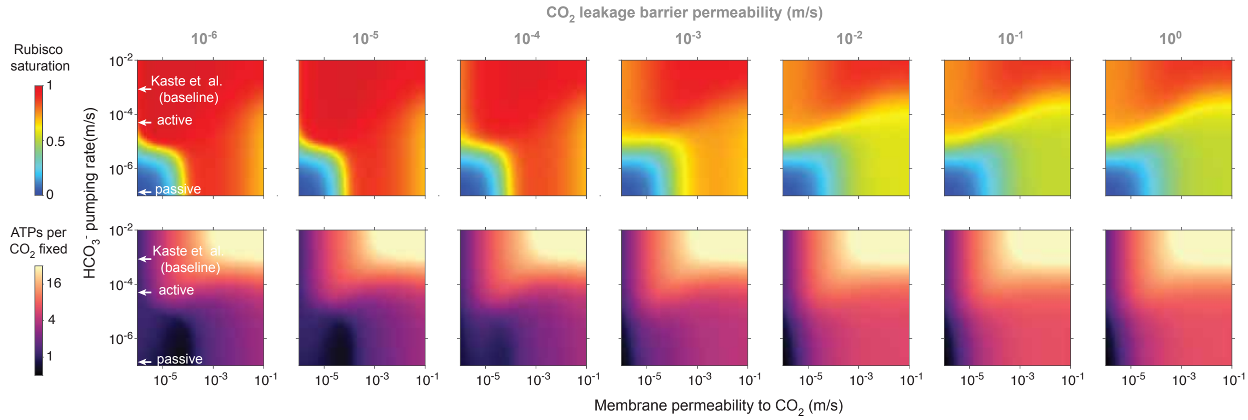

Supplementary Figure S1. The performance of a pyrenoid-based CO_2-concentrating mechanism depends on the permeability of the CO_2 leakage barrier around the pyrenoid. Heatmaps of (*top*) Rubisco saturation and (*bottom*) ATP per CO_2 fixed at varying membrane permeabilities to CO_2 and rates of bicarbonate pumping into the chloroplast are shown for CO_2 leakage barrier permeability of 10^{-6}, 10^{-5}, 10^{-4},10^{-3}, 10^{-2}, 10^{-1}, and 10^{0} m/s. Membrane permeability to CO_2 refers to the permeability of lipid bilayer membranes, including the chloroplast envelope and the thylakoid membranes. In contrast, CO_2 leakage barrier permeability represents the effective permeability of CO_2 diffusion barriers surrounding the pyrenoid.

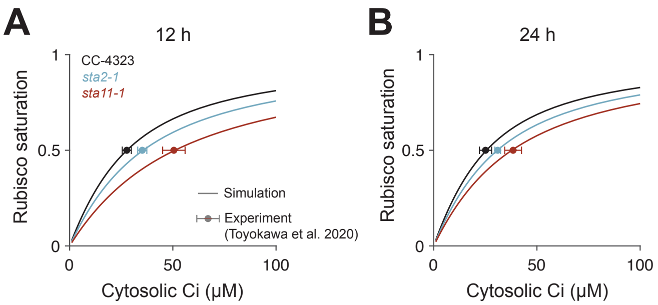

Supplementary Figure S2. Our model of the pyrenoid-based CO_2-concentrating mechanism is consistent with the inorganic carbon (Ci) affinity of wild-type Chlamydomonas cells and starch-deficient mutants measured in previous experiments. Toyokawa et al. (2020) measured the Ci affinity of three strains at pH 7.8: the wild-type strain CC-4323 and the starch-deficient mutant strains, *sta2-1*and *sta11-1*. The CC-4323 cells have an intact starch sheath while the *sta2-1* cells have thinner starch plates than those in CC-4323 and the *sta11-1* cells essentially lack a starch sheath and only have a few small starch-granule-like structures at the pyrenoid periphery. Cells grown under high CO_2 level (~600 μM) were shifted to very low CO_2 level (~2 μM) for 12 or 24 h before measuring Ci affinities. To account for the CCM under very low CO_2, we consider the model shown in main Fig. 1C with bicarbonate pumping across the chloroplast envelope and a CO_2 leakage barrier around the pyrenoid. We fix the rate of bicarbonate pumping at 5×10^{-5} m/s and fit the permeability $k_{barrier}$ of the CO_2 leakage barrier to the experimental data. Rubisco saturation for varying Ci levels is shown for models fitted to **A**) the 12 h experiment, and **B**) the 24 h experiment. The fitted $k_{barrier}$ values are: **A**) $k_{barrier} = 0.9 \times 10^{-4}$ m/s for CC-4323, $k_{barrier} = 2.5 \times 10^{-4}$ m/s for *sta2-1*, $k_{barrier} = 6.5 \times 10^{-4}$ m/s for *sta11-1*, and **B**) $k_{barrier} = 0.5 \times 10^{-4}$ m/s for CC-4323, $k_{barrier} = 1.5 \times 10^{-4}$ m/s for *sta2-1*, $k_{barrier} = 3 \times 10^{-4}$ m/s for *sta11-1*. We note that, considering that the *sta11-1* mutant lacks a starch sheath, the $k_{barrier}$ values fitted for *sta11-1* cells presumably describe the effect of thylakoid membrane sheets as a CO_2 leakage barrier. Likewise, considering that CC-4323 has both thylakoid membrane sheets and a starch sheath, the $k_{barrier}$ values fitted for CC-4323 presumably describe the cumulative CO_2 leakage barrier effect of both thylakoid membranes and a starch sheath.

Supplementary Table S1. Simulation parameters for the model of the pyrenoid-based CO_2-concentrating mechanism.

Parameters	Fig. 1A,B	Fig. 1C,D	Fig. 2A,B	Fig. 2C,D	Fig. S2
HCO_3^- pumping rate across the chloroplast membrane	5×10^{-5} m/s	5×10^{-5} m/s	0 m/s	0 m/s	5×10^{-5} m/s
Reversibility of HCO_3^- pumping	0.01	0.01	1	1	0.01
Leakage barrier permeability to CO_2 and HCO_3^-	10^2 m/s	10^{-4} m/s	10^2 m/s	10^{-4} m/s	fitted
First-order rate of the stromal carbonic anhydrase	10^4 s^{-1}	0 s^{-1}	10^4 s^{-1}	10^4 s^{-1}	0 s^{-1}
Membrane permeability to CO_2	varied	varied	varied	varied	3×10^{-3} m/s

All other simulation parameters are the same as baseline values specified in Supplementary Table 2 of (Fei et al., 2022) unless otherwise specified.
